# Methods of Employing Electricity in Nervous Diseases

**Published:** 1895-11

**Authors:** Frederick Peterson

**Affiliations:** Chief of clinic, department for nervous diseases Vanderbilt clinic, College of Physicians and Surgeons, New York


### METHODS OF EMPLOYING ELECTRICITY IN NERVOUS DISEASES.

By FREDERICK PETERSON, M. D.,

Chief of clinic, department for nervous diseases Vanderbilt clinic, College of Physicians and Surgeons, New York.

IT HAS often seemed to the writer that within the compass of a short paper the guiding principles of electrical treatment in nervous diseases might be succinctly stated for the benefit of the general practitioner, who may be in a quandary often as exactly what particular method to employ in the cases he meets with in his daily practice. In the following abstract of a lecture are given, first, the general methods in use and then in alphabetical order an outline of the electrical treatment in each form of disease in which the neurologist is accustomed to employ this therapeutic agent.

### GENERAL CONSIDERATIONS.

For stimulation of nerves and contraction of muscles interruptions are made with the interrupting handle. When this is too stimulating and it is desired to diffuse the current through a part, an ordinary sponge electrode, without an interruptor, may be used just like a sponge, rubbing it up and down on the surface. This is called the *labile method.*

In most applications about the head, eyes, neck, spine, heart or other delicate organs, and in applications for the relief of pain, interruptions of the current are not desirable. The *stabile method* is then used, *i. e.*, the cathode being on some indifferent part, the anode is fixed over the seat of pain or over the spot requiring a sedative. Then the current is gradually turned on and, without interruption of any kind, raised to the strength required and as gradually reduced after a few minutes.

Where it is desired to stimulate nutrition in a part the galvanic current, of course, is used, and it is important and desirable to employ much larger electrodes than we are accustomed to use for this purpose. Thus, if it is wished to improve the nutrition in a leg affected by *poliomyelitis*, electrodes a foot or so in diameter should be applied to the atrophied parts and not the small-sized instruments we are so prone to employ. Better even than the electrodes of large area would be the immersion of the affected extremity in a water-bath, the water being made the anode or cathode as might seem most indicated. If this is adopted more generally we shall be in the future better rewarded for our efforts in such cases, and proof of the nutritive efficacy of the current will be abundantly presented. The roller electrode is very useful for this purpose.

In many works on electricity much is said about directional methods, the employment of ascending and descending currents upon the spine, for instance. The terms ascending and descending are quite useless. If the physician remembers merely the polar differences of the galvanic current his common sense will teach him where the electrodes are to be placed.

*General Faradisation and Galvanisation.*—Either of these may be accomplished by placing the electrodes in a wooden bath-tub, divided into two compartments by a rubber diaphragm containing an opening for the trunk of the body, or by having the patient sit upon or place his feet upon a large sponge electrode, or insert his feet into a foot-bath connected with one pole, the other pole being used to sponge the entire body and limbs. This method appeals chiefly to the mind by suggestion and is, therefore, useful in the *neuro-psychoses.* It has also probably some value because of its refreshing effects and with the galvanic current it may be used for the introduction of certain drugs, such as iodide of potassium and corrosive sublimate. As a rule, from five to fifteen minutes' application, three times a week or daily, is the length of time for employment in most neurological conditions.

### SPECIAL ELECTRO-THERAPEUTICS IN NEUROLOGY.

*Motor Disorders.*—We may treat paralysis and spasm by electricity. In paralysis the distinction between the two classes of disorders by the situation of the lesion in the cerebro-spinal, or the spino-muscular segment of the motor tract, is to be remembered. Since in the former the nerves and muscles react to faradaism and suffer no trophic disturbance, the indication for electricity is almost entirely to assist in the prevention of spasmodic contractures. Hence the exercise of the rigid muscles by faradaism is useful. The other form of paralysis, which we may call peripheral, requires faradaism, if the affection is so mild that the muscles still react to this current. Galvanism is also useful in addition because of its nutritive effects upon the partly degenerated nerve and muscle. If, however, the peripheral paralysis is severe, faradaism is of no value, since the muscles cannot be exercised by it, and it has little or no effect upon nutrition. Here galvanism should be used, not only for its value as a trophic agent, but also to exercise and strengthen the muscles by producing contractions.

Clonic spasm, such as tic convulsif and blepharospasm, may occasionally be improved by the use of the anode (best conjoined with cocaine by cataphoresis) over the affected muscles and nerves, the cathode being placed upon any remote part of the body, like the hand, back or breast.

Tonic spasm, such as torticollis and writer's cramp, may also be relieved to some degree by anodal galvanisation. In any form of spasm usually only the most recent cases are susceptible of mitigation or cure by electricity. In hysterical paralysis or spasms, strong faradisation and static electricity are of great value.

*Sensory Disorders.*—An irritation or destructive lesion of any part of the sensory tract, from the cortex to the peripheral filaments, produces in the one case hyperesthesia (pain, neuralgia) and in the other anesthesia. Pain and neuralgia require anodal galvanisation and in pains or neuralgias of superficial nerves (trigeminal, intercostal, and the like,) the use of solutions of cocaine of from 10 to 20 per cent. with the anode is indicated. In all such applications for the relief of pain the electrode should be held steadily at one spot (the anode on the painful part, the cathode at any indifferent point,) and the current gradually diminished at the end of from ten to twenty minutes without interruption of any kind. Trigeminal, cervico-occipital, cervico-brachial, intercostal, lumbar, or sciatic neuralgia and rheumatic pains (like lumbago, headache and migraine,) are all to be treated upon this principle.

Anesthesia, on the contrary, is best alleviated by faradisation and the faradaic brush. The anesthetic area should be tapped with the brush with a current strong enough to be painful upon one's own hand. The skin should be dry and if necessary made more so by the use of chalk or powdered starch.

In organic diseases of the brain and spinal cord and in insanity, electricity is of doubtful service. Any value it may possess is probably due to psychic suggestion. But even such powerful suggestive agency is not to be despised. In some functional central diseases, such as neurasthenia, railway brain and railway spine (traumatic neuroses), hypochondriasis, and the like, general or local galvanisation and faradisation are often of considerable service.

CONDENSED LIST OF NERVOUS DISORDERS AND THE MODES OF APPLICATION OF ELECTRICITY WHERE IT IS INDICATED.

Abducens paralysis.—See *oculo-motor palsies.*

Abdominal neuralgia.—Stabile galvanisation from the back to the abdomen.

Amyotrophic lateral sclerosis.—Galvanism to the spine and to the atrophied muscles.

Anesthesias.—The faradaic brush with the current strong enough to redden the skin.

Asphyxia.—The faradaic brush to the epigastrium, neck, face and nostrils, or the interrupted faradaic current to the phrenic nerves.

Aphonia (hysterical).—Faradaism with one pole on each side of the larynx.

Ataxic paraplegia.—Galvanism to the spine; faradaism to exercise rigid muscles.

Basedow's disease.—Stabile galvanisation with the anode over the great nerves in the side of the neck and the cathode between the shoulders.

Blepharospasm.—Anodal galvanisation at the outer corner of the eye (10 per cent. cocaine cataphoresis sometimes useful).

Brain, organic disease of.—Electricity useless, except a mild galvanic current only and without interruptions as a *placebo.*

Bulbar paralysis.—Faradaism or galvanism as needed to contract the muscles of the tongue, lip and pharynx.

Bell's palsy.—See neuritis.

Catalepsy.—The faradaic brush to the surface of the body; phrenic faradisation.

Cephalalgia.—Stabile galvanisation of the head; the static breeze.

Cervico-brachial neuralgia.—Stabile anodal galvanisation over the painful nerves, the cathode on the back or in the hand.

Chorea.—Faradaism useful to exercise the muscles, which are sometimes paretic, but only after the choreic movements have ceased..

Coccygodynia.—Stabile anodal galvanisation at the coccyx with the cathode on the abdomen.

Contractures.—Faradisation of motor points to exercise the rigid muscles and especially their opponents.

Deltoid paralysis (circumflex nerve).—Faradaism to exercise the muscle.

Depression, mental.—General faradisation.

Dyspepsia, nervous.—General faradisation and faradaic baths.

Diphtheritic paralysis.—Exercise of muscles with the faradaic or galvanic current, as required, at motor points.

Enteralgia.—See *abdominal neuralgia.*

Exophthalmic goitre.—See *Basedow's disease.*

Enuresis nocturna.—Sometimes relieved by vesical faradisation with one electrode on the symphysis, or one urethral electrode introduced into the bladder, the other applied to the sacrum ; moderate current with interruptions.

Erb's paralysis.—Faradisation or galvanisation, according to the reaction, at motor points, to exercise the affected muscles.

Facial paralysis.—See *neuritis.*

Facial tic.—See *spasm.*

Gastralgia.—Anodal galvanisation of the epigastrium with the cathode on the back.

Genital neuroses and psychoses.—General faradisation.

Hallucinations.—Galvanism and faradaism have been known, in a few cases, to stop hallucinations of sight and hearing, probably by suggestion ; the poles to be applied to the affected organs.

Headache.—See *cephalalgia.*

Hemicrania.—See *migraine.*

Hemiplegia.—Faradaism to exercise the muscles and prevent contractures ; the faradaic brush in the presence of hemianesthesia.

Hypochondriasis.—General faradisation.

Hysteria.—Faradaism in paralysis and contractures ; the faradaic brush in anesthesia ; general galvanisation sometimes useful ; the static current for suggestion.

Locomotor ataxia.—Galvanisation of the spine employed usually, but of no actual benefit ; anesthesia to be treated with the faradaic brush ; lightning pains with the galvanic anode (occasionally cocaine cataphoresis).

Lumbago.—The faradaic brush for counter-irritation ; the galvanic anode to the painful region.

Melancholic stupor.—General faradisation.

Migraine.—Galvanism with one pole on each mastoid process ; stabile, from five to ten minutes.

Mastodynia.—Anodal galvanisation of the painful spot, the cathode on indifferent point.

Multiple spinal sclerosis.—Galvanism is used, but is of no actual service.

Musculospinal paralysis.—Faradisation of motor points if the extensors react; otherwise galvanism, with the cathode to contract the muscles and improve nutrition.

Myelitis.—Galvanism of the spine; faradaism to spastic muscles; the faradaic brush for anesthesias; the galvanic anode for pain; the cathode or anode for atrophied muscles.

Neuritis, multiple, alcoholic, arsenical, lead, meta-diphtheritic, rheumatic, and the like.—If the muscles react to faradaism, exercise them with that current; if not, use galvanism; labile galvanisation to improve nutrition.

Neuralgia.—The faradaic brush is occasionally useful over the painful region, but the stabile galvanic anode is the best, and when superficial nerves are affected, cocaine cataphoresis (a 10 to 20 per cent. solution).

Neurasthenia.—General faradisation or galvanisation, or the static current for suggestive purposes.

Neuroma.—When superficial, cocaine cataphoresis.

Oculo-motor palsies.—A sponge anode over the eyeball; mild stabile galvanisation.

Pianist's cramp.—See *professional neuroses.*

Phrenic-nerve stimulation.—Both sponge electrodes to the two phrenic-nerve points, or one over the nerve and one at the epigastrium; faradaic current, interrupted every two or three seconds.

Pleurodynia.—Stabile galvanisation with the anode to the painful point, or the faradaic brush to counterirritate.

Poliomyelitis.—Labile galvanisation to improve the nutrition of atrophic muscles and to exercise them.

Progressive muscular atrophy.—Galvanism to improve the nutrition and exercise the atrophied muscles.

Professional neuroses (writer's, pianist's, compositor's, and the like).—Faradaism to exercise the muscles; galvanism to improve the nutrition.

Pseudo-hypertrophic paralysis.—Local galvanisation and faradisation of muscles.

Ptosis.—See *oculo-motor palsies.*

Sacral neuralgia.—See *neuralgia.*

Sciatica.—A large, flat sponge anode over the painful points, with the cathode as far away as possible; stabile galvanisation; a steady, strong current, without interruption, for fifteen minutes daily in recent cases. In old cases electricity is of little value.

Spasm.—Electrical treatment of the cause (neuritis, neurasthenia, hysteria, peripheral irritations, and the like); the galvanic anode to the nerve involved and over painful points. Cocaine cataphoresis is useful in superficial nerve spasms (blepharospasm, facial tic, and the like).

Spinal cord diseases.—Spinal galvanisation ; a steady current, as strong as can be borne (from ten to fifteen milliampères), without interruption ; either pole at the nape of the neck and the other on the lumbar region. The distinction between ascending and descending currents in the treatment of chronic diseases of the spinal cord, is altogether absurd. The only value of electrical treatment in such cases is probably psychic.

Syncope.—See *asphyxia.*

Syringomyelia.—The faradaic brush to anesthetic areas ; galvanism to exercise and improve nutrition in atrophied muscles. See also *spinal cord diseases.*

Spinal irritation.—The faradaic brush along the spine ; general faradisation.

Singultus.—Faradisation of the phrenic nerve. See also *asphyxia.*

Tabes.—See *locomotor ataxia* and *spinal cord diseases.*

Telegrapher's cramp.—See *professional neuroses.*

Third-nerve paralysis.—See *oculo-motor paralysis.*

Tic douloureux.—See *neuralgia.*

Torticollis.—Labile anodal galvanisation of the affected muscle.

Trigeminal neuralgia.—The anode over the painful point (with a 10 to 20 per cent. solution of cocaine) ; the cathode between the shoulders or in the hand ; no interruption ; fifteen minutes ; fifteen milliampères. See also *neuralgia.*

Vesical paralysis.—See *enuresis nocturna.*

Vomiting (hysterical and nervous).—Stabile anodal galvanisation of the epigastrium, with the cathode on the back.

Wrist-drop.—See *neuritis* and *musculo-spinal paralysis.*

Writer's cramp.—See *professional neuroses.*

Wryneck.—See *torticollis.*

60 West 50th Street.

---

Drugs Contra-Indicated During Pregnancy.—At the head of dangerous substances (*Medical Age*) for the pregnant woman may be placed sodium salicylate, ergot, salicylic acid, and salol ; then come purgatives, antipyrin, acetanilid, sulphonal and cocaine.—M. Huguenin.

